# Improved production of a recombinant *Rhizomucor miehei* lipase expressed in *Pichia pastoris* and its application for conversion of microalgae oil to biodiesel

**DOI:** 10.1186/1754-6834-7-111

**Published:** 2014-08-04

**Authors:** Jinjin Huang, Ji Xia, Zhen Yang, Feifei Guan, Di Cui, Guohua Guan, Wei Jiang, Ying Li

**Affiliations:** State Key Laboratories for Agro-biotechnology and College of Biological Sciences, China Agricultural University, 2#,Yuanmingyuan West Road, Beijing, 100193 China; Key Laboratory of Human Disease Comparative Medicine, Ministry of Health, Institute of Laboratory Animal Science, Chinese Academy of Medical Sciences & Comparative Medical Center, Peking Union Medical College, 5#, Panjiayuannanli Street, Beijing, 100021 China

**Keywords:** *Rhizomucor miehei*, Lipase, Heterologous expression, *Pichia pastoris*, Microalgae oil, Biodiesel

## Abstract

**Background:**

We previously cloned a 1,3-specific lipase gene from the fungus *Rhizomucor miehei* and expressed it in methylotrophic yeast *Pichia pastoris* strain GS115. The enzyme produced (termed RML) was able to catalyze methanolysis of soybean oil and showed strong position specificity. However, the enzyme activity and amount of enzyme produced were not adequate for industrial application. Our goal in the present study was to improve the enzyme properties of RML in order to apply it for the conversion of microalgae oil to biofuel.

**Results:**

Several new expression plasmids were constructed by adding the propeptide of the target gene, optimizing the signal peptide, and varying the number of target gene copies. Each plasmid was transformed separately into *P. pastoris* strain X-33. Screening by flask culture showed maximal (21.4-fold increased) enzyme activity for the recombinant strain with two copies of the target gene; the enzyme was termed Lipase GH2. The expressed protein with the propeptide (pRML) was a stable glycosylated protein, because of glycosylation sites in the propeptide. Quantitative real-time RT-PCR analysis revealed two major reasons for the increase in enzyme activity: (1) the modified recombinant expression system gave an increased transcription level of the target gene (*rml*), and (2) the enzyme was suitable for expression in host cells without causing endoplasmic reticulum (ER) stress. The modified enzyme had improved thermostability and methanol or ethanol tolerance, and was applicable directly as free lipase (fermentation supernatant) in the catalytic esterification and transesterification reaction. After reaction for 24 hours at 30°C, the conversion rate of microalgae oil to biofuel was above 90%.

**Conclusions:**

Our experimental results show that signal peptide optimization in the expression plasmid, addition of the gene propeptide, and proper gene dosage significantly increased RML expression level and enhanced the enzymatic properties. The target enzyme was the major component of fermentation supernatant and was stable for over six months at 4°C. The modified free lipase is potentially applicable for industrial-scale conversion of microalgae oil to biodiesel.

**Electronic supplementary material:**

The online version of this article (doi:10.1186/1754-6834-7-111) contains supplementary material, which is available to authorized users.

## Background

A fungal lipase (*Rhizomucor miehei* lipase; RML) from *R. miehei* is a highly versatile biocatalyst used in laboratory and commercial/ industrial applications [1]. RML is a single-chain α/β type protein, MW of approximately 31.6 kDa, comprised of 269 amino-acid residues [2]. Its high resolution three-dimensional structure has been determined, and the catalytic triad is Ser144, His257, Asp203 [3]. RML is synthesized as a precursor that includes a 70 amino-acid propeptide before the 269 amino-acid residues of the mature enzyme [4]. The crystal structure at 1.9 Å resolution and theoretical analysis of RML activation have been reported [3, 5].

Two forms of the enzyme (Palatase 2000 L in free form and Lipozyme RM IM in immobilized form) are currently commercially available from Novozymes (Novo Nordisk A/S Corp, Hillerod, Denmark) [6]. Because of its strong specificity, RML has been widely used in ester hydrolysis, ester synthesis, and transesterification reaction such as production of structured lipids [7], synthesis of butyl butyrate (pineapple flavor) [8], and synthesis of monoglyceryl esters from chiral and prochiral acid methyl esters [9]. Despite this wide applicability, cost remains a major obstacle to large-scale industrial use of RML [10]. Enhanced activity and reduced production cost of the enzyme are important goals.

*Pichia pastoris* is the most frequently used yeast system for heterologous protein production [11]. One advantage of this expression system is that its ability to perform eukaryotic post-translational modifications of heterologous proteins, such as glycosylation N-linked glycosylation is the most commonly when expressed heterologous proteins in *Pichia pastoris*, it was found that N-linked glycosylation also played an essential role in protein secretion [12, 13]. To date, over 350 recombinant proteins have been expressed in *P. pastoris* systems, with protein production of more than 10 g/L in some cases [14]. High-level expression in *P. pastoris* can be affected by factors such as nucleotide sequence properties, gene copy numbers, mRNA transcription, promoter choice, secretion signals, and protein folding in the endoplasmic reticulum (ER); these factors cannot be regulated solely through control of the fermentation process [15, 16]. Some studies have been successful in improving protein expression levels through regulation of the above factors [15, 17, 18].

There is increasing research and industrial interest in ‘biodiesel’ as a replacement for petroleum-derived diesel fuel [19]. Microalgae have many advantages as a source of biodiesel in comparison with food crops and non-food crops, and may be the only source that can be sustainably developed in the future [20, 21]. Previous studies have focused on the culture of microalgae and enhanced lipid content of microalgae cells [22–25], and conversion of microalgae oil to biodiesel [26–29]. The chemical conversion of microalgae oil to biodiesel using an acid or alkaline catalyst involves high energy consumption and pollution. Enzyme-catalyzed conversion reactions have obvious advantages and are a ‘hot’ research topic. Various studies have attempted to improve reaction efficiency through the discovery of new types of enzymes [30], molecular enzyme modification [31–33], and reaction process optimization [34, 35].

We previously cloned the gene of 1,3-specific RML from *R. miehei* and expressed it in *P. pastoris* strain GS115 (RML-GS115). The enzyme was used to catalyze methanolysis of soybean oil and displayed strong positional specificity. However, the enzyme activities obtained were only 50 U/mL in a flask with medium Buffered Glycerol-complex Medium (BMGY) and Buffered Methanol-complex Medium (BMMY) and 550 U/mL in a 7.5-L fermenter [34]. In order to increase RML activity and obtain recombinants with maximal expression capacity, we considered factors such as propeptide, codon usage of signal peptide, and gene dosage of target protein in the present study. We significantly enhanced enzyme activity by changing the expression system to *P. pastoris* strain X-33 and vector pPICZaA, adding one propeptide to RML, and selecting the optimal gene dosage.

## Results

### Addition of the propeptide of the target gene

**Figure 1:**
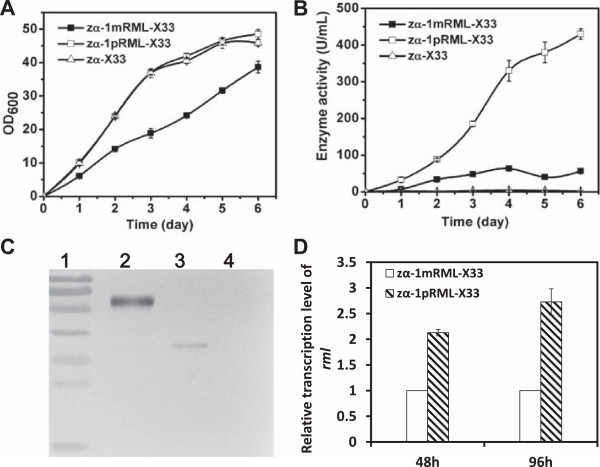
**Effect of propeptide addition on cell growth and target enzyme activity. A**: OD_600_ of recombinant strains in flask fermentation. Cell growth of control strain (zα-X33) and recombinant strain (zα-1pRML-X33) containing the propeptide sequence was much higher than that of the recombinant strain without the propeptide sequence (zα-1mRML-X33). **B**: Enzyme activity of recombinant strains in flask fermentation. Extracellular enzyme activity of zα-1pRML-X33 (430 U/mL) was 7.7-fold higher than that of zα-1mRML-X33 (56 U/mL). **C**: Extracellular protein production in fermentation supernatant detected by Western blotting. Lane 1: protein markers (top to bottom: 100, 70, 55, 40, 35, 25, 15 kDa). Lane 2: zα-1pRML-X33. Lane 3: zα-1mRML-X33. Lane 4: zα-X33. Each sample tested was 10 μL of 10 × diluted fermentation supernatant. The concentration of secreted extracellular target protein for zα-1pRML-X33 (0.15 mg/mL) was higher than for zα-1mRML-X33 (0.019 mg/mL). **D**: Comparison of the transcription level (by qPCR) of *rml* in zα-1pRML-X33 vs. zα-1mRML-X33. When the same signal peptide codons were present in the expression plasmid, adding the propeptide resulted in upregulation of target gene expression at both 48 and 96 hours.

We explored several strategies to modify RML and its heterologous expression system in order to improve enzyme activity and properties. Our first step was to examine the role of the target gene’s propeptide. We constructed two expression plasmids with carrier pPICZαA: one adding the target gene’s 70-amino-acid propeptide (*prml*) and one without propeptide (*mrml*). The recombinant plasmids were transformed separately into strain X-33. The positive strains, each containing one copy of the target gene, were termed zα-1mRML-X33 and zα-1pRML-X33. These two strains and a control strain (zα-X33, without target gene) were cultured in shaking flasks and sampled every day for cell growth and lipase activity. The screening results are shown in Figure 1. Cell growth of zα-1pRML-X33 and zα-X33 was higher than that of zα-1mRML-X33 (Figure 1A). The extracellular enzyme activity of zα-pRML-X33 (430 U/mL) was 7.7-fold higher than that of zα-1mRML-X33 (56 U/mL; Figure 1B). These findings indicate that in the absence of the propeptide, expression levels of the heterologous protein are consistent regardless of whether strain GS115 or X-33 is used as a host. Extracellular protein in fermentation supernatant was detected by Western blotting (Figure 1C). The content of secreted target protein for zα-1pRML-X33 (0.15 mg/mL) was approximately 7-fold higher than that for zα-1mRML-X33 (0.019 mg/mL).Quantitative real-time RT-PCR (qPCR) results confirmed that adding the propeptide resulted in upregulation of target gene expression (Figure 1D) at 48 and 96 hours. The propeptide evidently plays a promoting role in lipase activity and heterologous protein secretion.

### Optimization of the signal peptide codons

**Figure 2:**
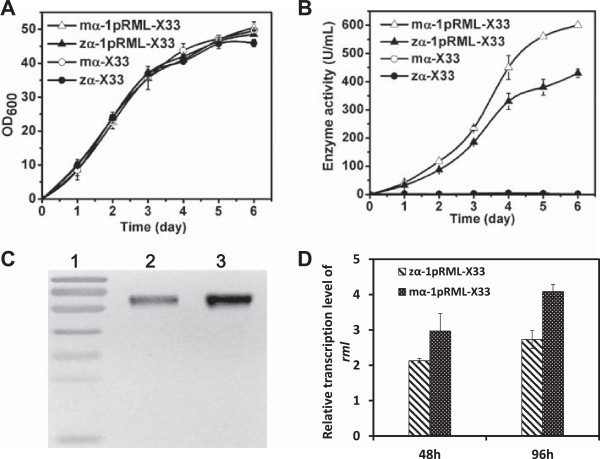
**Cell growth and protein production of recombinant strain with modified α-factor signal peptide (mα-1pRML-X33), strain with original α-factor signal peptide (zα-1pRML-X33), and two control strains (mα-X33 and zα-X33). A**: OD_600_ of the recombinants in flask fermentation. Cell growth did not differ notably among the four strains. **B**: Enzyme activity of the recombinants during fermentation. Activity of mα-1pRML-X33 (600 U/ml) was higher than that of zα-1pRML-X33 (430 U/ml). Activity increased 1.4-fold after optimization of signal peptide codons. **C**: Extracellular protein expression in fermentation supernatant detected by Western blotting. Lane 1: protein markers (same as in Figure 1). Lane 2: recombinant strain containing original α-factor signal peptide. Lane 3: strain containing modified α-factor signal peptide. The concentration of extracellular target protein for mα-1pRML-X33 (0.22 mg/mL) was higher than that for zα-1pRML-X33 (0.16 mg/mL). **D**: The transcription level of *prml* (detected by qPCR) in mα-1pRML-X33 vs. zα-1pRML-X33. Optimization of signal peptide (mα-1pRML-X33) increased target gene transcription at both 48 and 96 hours.

The original expression plasmid (pPICZαA) and the modified signal peptide plasmid (pPICMαA) were kindly provided by Drs Yijun Huang and Jia Ban. We compared their effects on target enzyme activity and protein secretion. The nucleotide sequence of the modified signal peptide (mα) is shown in Additional file 1: Figure S1. We successfully constructed modified codons of the signal peptide recombinant strain (termed mα-1pRML-X33) and the original recombinant strain (termed zα-1pRML-X33). Each strain contained one copy of the target gene. Flask fermentation of the two strains is compared in Figure 2. Cell growth was essentially the same in mα-1pRML-X33, zα-1pRML-X33, and two control strains without the target gene (mα-X33, zα-X33); they all reached OD_600_ ≈ 50 (Figure 2A). However, the enzyme activity of mα-1pRML-X33 (600 U/mL) was higher than that of zα-1pRML-X33 (430 U/mL; Figure 2B). Optimization of the signal peptide codons resulted in a 1.4-fold increase in enzyme activity. The target protein was also detected by Western blotting. The amount of extracellular target protein (0.22 mg/mL) from mα-1pRML-X33 (Figure 2C, lane 3) was higher than that (0.16 mg/mL) from zα-1pRML-X33 (lane 2). Target gene transcription level was evaluated by qPCR. For a given dosage of the target gene in the cells, optimization of the signal peptide clearly increased enzyme activity and the amount of extracellular target protein. The transcription level of the target gene was 1.4-fold higher following optimization of the signal peptide codons (Figure 2D). These findings indicate that optimization of codons in the signal peptide to make it more suitable for transcription in host cells promoted expression and secretion of target heterologous protein.

### Optimization of the target gene dosage

We next investigated the effects of target gene copies on protein expression. We successfully constructed recombinants with 1, 2, 4, and 8 copies of the *prml* gene, termed respectively as mα-1pRML-X33, mα-2pRML-X33, mα-4pRML-X33 and mα-8pRML-X33. These recombinants were tested by qPCR. The standard curves of glyceraldehyde-3-phosphate dehydrogenase gene (*gap*; used as a reference gene) and *rml* detected by qPCR are shown in Additional file 2: Figure S2. C(t) values of the target gene (*prml*) and reference gene (*gap*) and copy numbers were calculated using the formula given in the Materials and Methods section ‘Screening for target transformants’, and results are shown in Additional file 3: Table S1.

**Figure 3:**
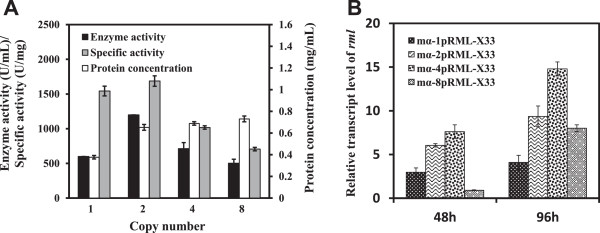
**Comparison of recombinant strains containing 1, 2, 4, and 8 gene copies of** ***prml.*** **A**: After 96 hours incubation at 30°C, mα-2pRML-X33 displayed higher enzyme activity (1200 U/mL), specific activity (1684 U/mg), and protein concentration (0.770 mg/mL) in the fermentation supernatant. **B**: *rml* transcription level in the 1-, 2-, 4, and 8-copy strains. At both 48 and 96 hours, transcription level was highest in the 4-copy strain and lower in the 8-copy strain.

The differential effects of the four recombinants on enzyme activity in flask fermentation are summarized in Figure 3A. When the target gene copy number was increased from 1 to 2, enzyme activity was doubled (from 600 to 1,200 U/mL) and extracellular protein content also increased approximately 2-fold (from 0.38 to 0.770 mg/mL). When copy number increased from 4 to 8, extracellular protein content was essentially unchanged (0.69 versus 0.73 mg/mL) and enzyme activity decreased from 713 to 503 U/mL. As copy number increased from 1 to 2, enzyme specific activity increased slightly from 1543 to 1684 U/mg, and then decreased for copy number 4 (1018 U/mg) and 8 (707 U/mg). The lower proportion of target protein in total protein may account for the reduced enzyme activity and specific activity for target gene copy numbers 4 and 8 in comparison with copy number 2. These findings indicate that 2 is the optimal copy number for *prml* expression in strain X-33. The 2-copy recombinant strain had the highest enzyme activity, extracellular protein content, and specific activity. Although qPCR analysis showed the highest target gene transcription level for the 4-copy strain (Figure 3B), our experiments showed that this strain did not have the maximal enzyme activity. Possible reasons for the advantage of the 2-copy strain over the 4-copy or 8-copy strain in terms of protein modification, transport pathways, and unfolded protein response (UPR)-related genes are considered in the ‘Discussion’ section. In conclusion, the lipase with maximal enzyme activity was secreted by the 2-copy strain (mα-2pRML-X33). This enzyme was termed Lipase GH2 and used in subsequent experiments.

### Enzymatic characterization of Lipase GH2

#### Detection of glycosylation

Bioinformatics analysis indicated glycosylation sites in the propeptide region of the target gene. We therefore evaluated glycosylation level of the protein. zα-mRML-X33 (mRML) and mα-2pRML-X33 (pRML; Lipase GH2) were selected for the deglycosylation reaction. The extracellular target protein was analyzed by Western blotting. Because of the addition of its propeptide a broad molecular weight (MW) of 55 to 70 kDa was found for Lipase GH2 (Additional file 4: Figure S3, lane 2). Deglycosylation of Lipase GH2 by Endo Hf resulted in MW of approximately 43 kDa (Additional file 4: Figure S3, lane 3). The same MW is seen in lanes 4 and 5 of Additional file 4: Figure S3 because the enzyme (mRML) lack the propeptide; there is no effect on MW regardless of the deglycosylation treatment for the proteins.

#### Enzyme properties

Various properties (optimal temperature and pH, temperature and pH tolerance, methanol and ethanol tolerance) were compared among two of the newly constructed strains (mα-mRML-X33, mα-2pRML-X33) and a previously constructed strain (RML-GS115). The results are shown in Table 1. The most striking difference was the lower optimal temperature of Lipase GH2 (35°C) as compared with mRML (45°C). Lipase GH2, in comparison with mRML, also had approximately 2-fold higher values of temperature tolerance (0 to 70°C versus 0 to 40°C), methanol tolerance (0 to 40% versus 0 to 20%, v/v), and ethanol tolerance (0 to 20% versus 0 to 10%, v/v). These findings indicate adding the propeptide caused glycosylation that results in improved enzyme thermostability and ability to tolerate adverse environmental factors.

**Table 1:** **Enzymatic properties of** ***R. miehei*** **lipase expressed in three forms**

	Optimal temperature (°C)	Temperature tolerance (°C)	Optimal pH	pH tolerance	Methanol tolerance (%)	Ethanol tolerance (%)
Lipase GH2 (X-33)	35	0-70	6.0	4-10	0-40	0-30
mRML (X-33)	45	0-40	8.6	4-10	0-20	0-10
RML (GS115)	50	0-50	8.0	4-9	0-30	0-20

### Conversion of microalgae oil to biodiesel using Lipase GH2

Microalgae (*Chlorella vulgaris*) powder was kindly provided by Dr Peng Pu (State Key Laboratory of Catalytic Material and Reaction Engineering, Research Institute of Petroleum Processing, SINOPEC, Beijing, China). We extracted total lipids from the powder by the method of Tran *et al*. [36] and analyzed the fatty acid composition by gas chromatography [37]. Results are shown in Additional file 5: Table S2. The microalgae oil extracted from 1 g microalgae powder contained 508.26 mg fatty acids, of which C16 to C18 fatty acids comprised 487.52 mg (96% of the total). Therefore, microalgae oil is a potentially useful substrate for preparation of biodiesel.

We previously described a practical design for the conversion of soybean oil to biodiesel using a combination of two enzymes [34]. In the present study, we used two treatment strategies for the conversion of microalgae oil to biodiesel using Lipase GH2: (1) Lipase GH2 as a catalyst; (2) combined catalysis using two enzymes (Lipase GH2 and a non-specific mono/diacylglycerol lipase (MDL)) in a single reaction. The reaction conditions are described in the ‘Materials and Methods’ section. Gas chromatography (GC) and thin-layer chromatography (TLC) were used for product detection.

**Figure 4:**
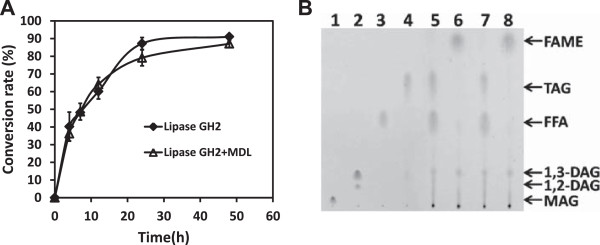
**Detection of esterification reaction products by GC and TLC. A**: GC detection. The substrate (microalgae oil) more than 90% conversion to biodiesel (FAME) after esterification reaction for 24 hours at 30°C. There was no significant difference between reactions catalyzed by a single lipase (Lipase GH2) vs. a combination of two lipases (Lipase GH2 and MDL). **B**: TLC detection. Lane: 1-monooleate (MAG). Lane 2: dioleoylglycerol (1,3- and 1,2-diacylglycerol). Lane 3: oleic acid (FFA). Lane 4: triacylglycerol (TAG). Lanes 5–6: single-lipase (Lipase GH2) treatment at 0 h and 24 h. Lanes 7–8: two-lipase (Lipase GH2 and MDL) treatment at 0 and 24 hours.

Substrate changes detected by GC at various times during methanolysis reaction of microalgae oil are shown in Figure 4A. For treatments 1 and 2 as above, substrate conversion rates after 24 hours reaction at 30°C were 91% and 89%, respectively. Most of the substrate (microalgae oil) was converted to biodiesel, and there was no significant difference between treatments 1 and 2.

Similar results were obtained for each sample by TLC (Figure 4B). Standard samples (shown in lanes 1 to 4) were monoacylglycerol (MAG), diacylglycerol (DAG), free fatty acid (FFA) and triacylglycerol (TAG). Fatty acids are the major components of microalgae oil. Substrates (FFA and TAG) prior to esterification reaction are shown in lanes 5 and 7. After the final reaction (24 hours at 30°C), the substrates were almost completely converted to fatty acid methyl esters (FAME; lanes 6 and 8), and there was no significant difference between treatment 1 (lanes 5 and 6) and treatment 2 (lanes 7 and 8).

In conclusion, Lipase GH2 can be used as an effective catalyst for the conversion of microalgae oil to biodiesel with no requirement for a second enzyme as an auxiliary factor.

## Discussion

### Adding propeptide induced protein glycosylation and improved enzyme tolerance

Heterologous protein expression is affected by numerous factors, including the expression vector, host strain, gene structure, signal peptide, gene dosage, and culture conditions for recombinants [38, 39]. In this study, we successfully constructed a high-expressing recombinant strain through propeptide addition, optimized codon usage of the signal peptide, and optimized gene dosage. Glycosylation has been shown to promote enzyme stability [40]. In the case of subtilisins, the 77-residue propeptide acts as an intramolecular chaperone that organizes the correct folding of its own protease domain; the occurrence of a mutation (Ile (-48)-to-Val) in the propeptide results in two subtilisins differing in secondary structure, thermostability, and substrate specificity [41]. Based on these previous findings, we hypothesized that the 70-residue propeptide of *R. miehei* lipase (RML) may also have affected the enzyme folding or post translational modification. An advantage of *P. pastoris* as an expression system is its ability to undergo eukaryotic post-translational modifications, including glycosylation of heterologous proteins [12]. We found that the broad MW of Lipase GH2 observed on Western blotting is a result of glycosylation. Amino acid sequence analysis revealed two potential N-glycosylation sites (Asn-Xaa-Ser/Thr), both located in the propeptide sequence; they were identified by NetNGlyc 1.0 Server analysis (http://www.cbs.dtu.dk/services/NetNGlyc) at the 8^th^ position with the NST(Asn-Ser-Thr) motif and the 58^th^ position with the NAT(Asn-Ala-Thr) motif. Glycosylation enhanced the ability of Lipase GH2 to tolerate temperature, methanol, and ethanol, and to catalyze the enzymatic conversion of microalgae oil to biodiesel. Thus, in the case of Lipase GH2, addition of heterologous gene propeptide provides a dual benefit: it enhances enzyme tolerance and increases enzyme activity.

### Why are two target gene copies better than four copies?

In secretory pathways, cells use specialized quality control systems to ensure that only properly folded and modified proteins are secreted into the extracellular space; unfolded or incorrectly folded (misfolded) proteins are reverse-transported to the cytoplasm by the ER degradation system or the lysosomal degradation system [42]. Correct folding of heterologous proteins is crucial in that it determines enzyme activity and functions.

How does integration of different numbers of target gene copies in recombinant strains affect protein transcription levels, folding, and secretion? We used qPCR detection to study expression of genes previously reported to be involved in protein folding, modification, and translocation pathways in yeast (see Additional file 6: Table S3). We focused on *prml* gene expression to examine the effect of target gene copy number.

We observed an association of four unfolded protein response (UPR)-related genes that have been described previously [43]. *PDI*, located in the ER membrane, encodes protein disulfide isomerase which is responsible for protein disulfide bond formation. *ERO1,* located in the ER membrane, is an ‘assistant’ of *PDI*, responsible for the glycoprotein required for oxidative protein folding in the ER. *KAR2,* located in the ER lumen, is a major Hsp70 chaperone. *HAC1*, located in the nucleus, is a transcription activator that regulates the UPR via UPRE (UPR element) binding and membrane biogenesis. Stress to the ER triggers increased expression of these genes and possible relief of the stress through the gene products.

**Figure 5:**
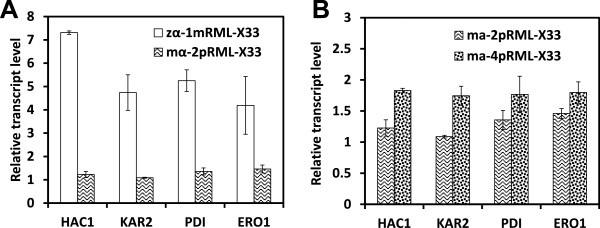
**Transcription levels of four UPR-related genes in different recombinants. A**: Transcription levels of *HAC1*, *KAR2*, *PDI*, and *ERO1* were lower in mα-2pRML-X33 and higher in zα-1mRML-X33. **B**: Transcription levels of four UPR-related genes in the 2-copy vs. 4-copy strains. The transcriptional levels of four genes were all higher in 4-copy strain than that of 2-copy strain.

Results for detection of these genes in strains zα-1mRML-X33 (not optimized) and mα-2pRML-X33 (optimized) are shown in Figure 5A. Transcriptional levels of all four genes were higher in zα-1mRML-X33 than that in mα-2pRML-X33. These findings suggest that some mRMLs may be expressed with abnormal structure or misfolded proteins, thereby inducing ER stress and initiating UPR.

After finding that enzyme activity and target gene transcription level were lowest for the 1-copy and 8-copy recombinant strains (see Results - ‘Optimization of the target gene dosage’), we compared target gene expression in the 2-copy and 4-copy strains (Figure 5B). In the 4-copy strain, the transcriptional levels of four UPR-related genes were all higher than that of 2-copy strain, it seems that protein folding generated obstacles and was unable to improve enzyme activity or protein secretion. Thus, although the 4-copy strain (mα-4pRML-X33) had a higher transcription level of the target gene than did the 2-copy strain, the abnormal protein resulted in ER stress, abnormal protein assembly and modification, and triggering of UPR (upregulation of UPR-related genes). However, the UPR did not relieve the ER stress. These findings illustrate why 2 target gene copies in the host are better than 4 copies.

It is important to note that there was no positive correlation between gene dosage and the target protein yield in construction of a heterologous protein expression system. Rather, the bearing capacity of the host cell must be considered. Abnormal protein folding and a variety of stress factors may interfere with protein polymerization in the ER lumen, leading to accumulation of unfolded or misfolded proteins and consequent ER stress [44–46]. If the stress is too high, it cannot be relieved by upregulation of UPR-related genes.

### Lipase GH2 is a useful catalyst for biodiesel synthesis

Lipase can be used in free or immobilized form in the biodiesel preparation process. The immobilized form increases the chances of re-usability of biocatalysts, preserving enzyme activities over several reaction cycles and can be removed easily from the mixture and can be reused in future. While the free enzyme (fermentation supernatant) in reaction process can be directly used which to avoid the cost of immobilized enzyme [47–49]. We previously described two successful methods for conversion of soybean oil to biodiesel catalyzed by free lipase: (i) a two-step reaction using *Yarrowia lipolytica* lipase which can be used by 25 cycles to produce fatty acid ethyl ester [35], and (ii) a one-step reaction using a combination of RML and MDL to produce FAME [34]. Soybean oil consists primarily of TAG. The RML lipase (RML-GS115) is 1,3-specific and cannot break a two-ester bond. MDL is able to break a two-ester bond, and the two enzymes are able to cooperate in the transesterification reaction. Use of soybean oil as substrate in a reaction system for biodiesel synthesis involves both hydrolysis reaction (hydrolysis of fat into fatty acids and glycerol) and esterification reaction (synthesis of fatty acids and methanol into FAME). Use of the new expression system (mα-2pRML-X33) constructed in this study resulted in a more than 20-fold increase in target protein expression. The enzymatic properties of the new system (improved temperature, methanol, and ethanol tolerance) are more suitable for application in biodiesel preparation. Microalgae oil consists of FFA, TAG, DAG, and MAG, with FFA being the major component. Biodiesel preparation requires methyl esterification by lipase, for which the newly constructed Lipase GH2 is suitable. The glycosylated protein strongly retains its enzyme activity; when we kept the fermentation supernatant at 4°C and sampled the lipase activity twice per month, we found no reduction in the activity after six months. For microalgae oil as substrate, the fermentation supernatant (free lipase) functions as a direct catalyst in the esterification and transesterification reaction, and achieves good results. This design has the advantages of simplifying the production process, saving time, and avoiding the expense of immobilized enzyme.

The use of Lipase GH2 as a catalyst in methanolysis of microalgae oil for biodiesel synthesis is economical for industrial applications and has great potential. Our ongoing studies are focused on further improvement and optimization of enzyme quality, reaction conditions, reaction rate, and industrial applicability.

## Conclusions

Various strategies are available for improving the efficiency of heterologous protein production by *P. pastoris*. Integration of too many copies of the target gene in the host genome may lead to increased ER stress and reduced protein production. In this study, we significantly increased the RML expression level and modified enzymatic properties through optimal expression plasmid with signal peptide, addition of the target gene propeptide, and optimal exogenous gene dosage. Lipase GH2, the major component of the fermentation supernatant, is stable for over six months at 4°C and is an effective enzyme for catalytic conversion of microalgae oil to biodiesel. Further studies will lead to improved industrial productivity.

## Materials and Methods

### Gene cloning and construction of multimers *in vitro*

The *prml* and *mrml* genes were cloned from *R. miehei* lipase (GenBank accession number A02536.1) cDNA using two pairs of primers. *R. miehei* pro-mature lipase gene (*prml*) was amplified using the primer pairs pRML-f (5′-CCG*GAATTC*GTGCCAATCAAGAG-3′, *Eco*RI site) and RML-r (5′-CTAG*TCTAGA*GTACAGAGGCCTGTG-3′, *Xba* I site). Mature lipase gene (*mrml*) was amplified using the primer pairs mRML-f (5′-CCG*GAATTC*AGCATTGATGGTGG-3′, *Eco*R I site) and RML-r (5′-CTAG*TCTAGA*GTACAGAGGCCTGTG-3′, *Xba* I site). The italics indicate the enzymatic restriction sites. Two vectors were used: pPICZαA (Invitrogen; Carlsbad, California, United States), and pPICMαA (a pPICZαA-derived plasmid). The only difference between the two vectors is that the α-factor codons of pPICMαA are optimized, without change of the amino acid sequence. The sequences of the original and modified signal peptide were kindly provided by Dr Jia Ban; their differences are shown in Additional file 1: Figure S1. Single-copy recombinant plasmids pPICZa-1*prml*, pPICMa-1*prml*, and pPICZa-1*mrml* were constructed. pPICMaA plasmids containing various gene dosages (pPICMa-2*prml*, pPICMa-4*prml*, and pPICMa-8*prml*) were constructed as described by Menendez *et al*. [17]. The plasmids and strains used in this study are listed in Table 2.

**Table 2:** **Plasmids and strains used in this study**

	Description	Source
Plasmids		
pPICZα A	Secretion expression vector with α-factor from *S. cerevisiae*	Donated by Dr Yijun Huang
pPICMα A	pPICZα A with α-factor optimized codons	Donated by Dr Jia Ban
Strains		
X-33	Host strain (WT Mut^+^)	Donated by Dr Yijun Huang
zα-1mRML-X33	One copy mRML of the lipase without propeptide expressed in X-33 using pPICZα A	This study
zα-1pRML-X33	One copy pPML of the lipase with propeptide expressed in X-33 using pPICZα A	This study
mα-1pRML-X33	One copy pRML expressed in X-33 using pPICMα A	This study
mα-2pRML-X33	Two copies pRML expressed in X-33 using pPICMα A	This study
mα-4pRML-X33	Four copies pRML expressed in X-33 using pPICMα A	This study
mα-8pRML-X33	Eight copies pRML expressed in X-33 using pPICMα A	This study

### Screening for target transformants

The recombinant plasmids were transformed into X-33 and screened with Yeast Extract Peptone Dextrose Medium with Sorbitol (YPDS) medium as described by Hu *et al*. [50], except that the multi-copy recombinant plasmids could not be linearized before transformation into X-33 by electroporation. For each transformation, 50 positive transformants were selected by tributyrin flat screening. Eight strains were selected for each transformant based on the size of the transparent circle in fermentation. Positive transformants were flask-cultured in BMGY/BMMY medium as described by Hu *et al*. [50]. Every day, 1% methanol (Sinopharm Chemical Reagent Beijing Co., Ltd, Beijing, China) was used to induce protein expression and activity and production were measured for each strain.

Absolute quantification of target gene copies was performed by qPCR using SYBR Green I Mix (Roche, Mannheim, Germany). qPCR was conducted in a LightCycler 480 RT-PCR System(Roche, Mannheim, Germany) using LightCycler 480 SYBR Green I Master Kit (Roche). Specific primers purified by HPLC (Invitrogen) were designed to yield 100 to 250 bp products. All primers (*rml* and *gap*) used for qPCR are listed in Additional file 6: Table S3. Genomic DNA of *P. pastoris* was used as template. The genomic DNA of the single-copy strain was used to establish the standard curve (Additional file 2: Figure S2). The C(t) values of the target gene (*prml*) and reference gene (*gap*) are shown in Additional file 3: Table S1. The numbers of target gene copies were calculated by absolute quantification as described by Abad *et al*. [51], using the following formula:
1

### Transcription levels of genes related to various steps in protein expression, modification, and secretory pathways

There were a total of 14 genes related to the target lipase, UPR, protein transport, and protein secretion. RNA extraction and relative qPCR analysis were performed as described by Wang *et al*. [52]. The primers used for qPCR are listed in Additional file 6: Table S3.

### Western blotting and glycosyl chain detection

The recombinant lipase was tested by Western blotting as described by Shen *et al*. [16]. sodium dodecyl sulfate polyacrylamide gel electrophoresis (SDS-PAGE) was performed using 5% stacking gel and 12% resolving gel. Monoclonal mouse-anti-His-Tag antibody (Tiangen, Beijing, China) was used as the primary antibody and goat anti-mouse IgG antibody conjugated to alkaline phosphatase (Sigma-Aldrich, St. Louis, Missouri, United States) as the secondary antibody. The membrane was applied to a BCIP/NBT Chromogenic reagent kit (Tiangen) according to the manufacturer’s instructions.

Protein concentration was quantified by Bradford assay [53] using Bovine Serum Albumin (BSA) as a standard. Glycosyl chains in pRML were removed by Endo Hf (New England Biolabs; Beverly, Massachusetts, United States) according to the manufacturer’s instructions.

### Enzyme characterization

Lipase hydrolysis activity was measured as described in our previous study [35], except that we used the conditions 0.1 M sodium dihydrogen phosphate/citric acid, pH 6.0, 35°C for Lipase GH2 and 0.1 M CHES, pH 8.6, 45°C for mRML. Optimal pH values for Lipase GH2 and mRML were determined under standard conditions. The buffers used were 0.1 M sodium dihydrogen phosphate/citric acid (pH 3.12 to 8.17), 0.1 M Trihydroxymethyl aminomethane- hydrochloric acid (Tris-HCl) (pH 8.02-9.03), 0.1 M N-Cyclohexyl-2-aminoethanesulfonic acid (CHES) (pH 8.6-9.5), and 0.1 M N-Cyclohexyl-3-aminopropanesulfonic acid (CAPS) (pH 10.0-11.0). pH stability was determined at pH values ranging from 1.0 to 12.7 for 90 minutes, and residual activity was measured. Optimal temperatures were determined under standard conditions in the range 0 to 60°C. Thermostability was determined for 90 minutes at temperatures ranging from 0 to 80°C, and residual activity was measured.

### Biodiesel preparation and detection

Lipids were extracted from microalgae powder as described by Tran *et al*. [36], except that chloroform/methanol (1:2 v/v) (Sinopharm Chemical Reagent Beijing Co., Ltd, Beijing, China) was used as solvent. Oil extracted from microalgae powder was used as a substrate to produce biodiesel using the organic solvent n-hexane (Sinopharm Chemical Reagent Beijing Co., Ltd, Beijing, China). Production conditions were: 0.1 g of microalgae oil/30 μL methanol, 1 mL n-hexane, 55 μL Lipase GH2 (2-copy recombinant strain) (1200 U/mL) and 5 μL MDL (1500 U/mL) (for Lipase GH2 alone, MDL was replaced by H_2_O), incubation at 30°C and shaking at 150 rpm for 24 hours.

FAME in the reaction mixture were analyzed using a GC system (model 7890A, Agilent Technologies (Santa Clara, California, United States) equipped with a capillary column (CP-FFAP CB; 25 m × 0.32 mm × 0.3 μm; Agilent Technology). Sample preparation and detection conditions were as described by Ren *et al*. [54]. TLC was performed as in our previous study [35].

## Electronic supplementary material

Additional file 1: Figure S1: Sequence alignment of original signal peptide (zα) and modified signal peptide (mα) using DNAMAN program (Lynnon Corporation, United States). 46 codons in the modified signal peptide were optimized, resulting in changes of 50 nucleotides. G + C content increased from 41.1 to 47.0%. The modified signal peptide showed 82.5% identity with the original signal peptide (zα) after averaging the distribution of both the G + C content and the optimized codons. (PDF 186 KB)

Additional file 2: Figure S2: Standard curves of *gap* and *rml* detected by qPCR. (PDF 155 KB)

Additional file 3: Table S1: Target gene copy number in four recombinant strains. (PDF 144 KB)

Additional file 4: Figure S3: Detection of glycosylation of lipases. Deglycosylation treatment using Endo Hf showed that pRML (Lipase GH2) was a glycosylated protein, whereas mRML had no glycosylation. Lane 1: protein markers (top to bottom: 100, 70, 55, 40, 35, 25, 15 kDa). Lane 2: Lipase GH2 without Endo Hf (no deglycosylation). Lane 3: Lipase GH2 with Endo Hf (with deglycosylation). Lane 4: mRML with Endo Hf (with deglycosylation). Lane 5: mRML without Endo Hf (no deglycosylation). (PDF 112 KB)

Additional file 5: Table S2: Fatty acid analysis of microalgae oil by HPLC. (PDF 169 KB)

Additional file 6: Table S3: Genes and primers used for qPCR. (PDF 183 KB)

## Abbreviations

1: 2-DAG: 1,2-diacylglycerol
1: 3-DAG: 1,3-diacylglycerol
FAME: fatty acid methyl ester(s)
FFA: free fatty acid
MAG: monoacylglycerol
TAG: triacylglycerold.
