# Supplementary material for: Improved production of a recombinant Rhizomucor miehei lipase expressed in Pichia pastoris and its application for conversion of microalgae oil to biodiesel
Source: Biotechnol Biofuels. 2014 Aug 4;7:111. doi: 10.1186/1754-6834-7-111 (PMC4364654; doi:10.1186/1754-6834-7-111)
Supplement: Supplementary file 1 — Additional file 1: Figure S1: Sequence alignment of original signal peptide (zα) and modified signal peptide (mα) using DNAMAN program (Lynnon Corporation, United States). 46 codons in the modified signal peptide were optimized, resulting in changes of 50 nucleotides. G + C content increased from 41.1 to 47.0%. The modified signal peptide showed 82.5% identity with the original signal peptide (zα) after averaging the distribution of both the G + C content and the optimized codons. (PDF 186 KB) [file 13068_2014_511_MOESM1_ESM.pdf]

Additional files1： Figure S1

```
za.seq   TTATTCGAAACGATGAGATTTCCTTCAATTTTTACTGCTG     40
ma.seq   TTATTCGAAACGATGAGATTCCCATCTATTTTTACCGCTG     40
Consensusttattcgaaacgatgagatt cc tc atttttac gctg

za.seq   TTTTATTCGCAGCATCCTCCGCATTAGCTGCTCCAGTCAA     80
ma.seq   TTTTGTTCGCTGCCTCTTCCGCTTTGGCCGCTCCAGTCAA     80
Consensustttt ttcgc gc tc tccgc tt gc gctccagtcaa

za.seq   CACTACAACAGAAGATGAAACGGCACAAATTCCGGCTGAA    120
ma.seq   CACTACTACCGAGGACGAGACTGCTCAAATTCCTGCTGAG    120
Consensuscactac ac ga ga ga ac gc caaattcc gctga

za.seq   GCTGTCATCGGTTACTCAGATTTAGAAGGGGATTTCGATG    160
ma.seq   GCTGTCATCGGTTACTCTGACCTGGAAGGAGATTTCGACG    160
Consensusgctgtcatcggttactc ga  t gaagg gatttcga g

za.seq   TTGCTGTTTTGCCATTTTCCAACAGCACAAATAACGGGTT    200
ma.seq   TTGCTGTCTTGCCTTTCTCCAACTCCACCAACAATGGTTT    200
Consensusttgctgt ttgcc tt tccaac  cac aa aa gg tt

za.seq   ATTGTTTATAAATACTACTATTGCCAGCATTGCTGCTAAA    240
ma.seq   GTTGTTCATCAACACTACTATCGCTTCTATTGCTGCCAAG    240
Consensus ttgtt at aa actactat gc    attgctgc aa

za.seq   GAAGAAGGGGTATCTCTCGAGAAAAGAGAGGCTGAAGCTG    280
ma.seq   GAAGAGGGTGTTTCTCTTGAGAAAAGAGAGGCTGAAGCTG    280
Consensusgaaga gg gt tctct gagaaaagagaggctgaagctg

za.seq   AATTC                                       285
ma.seq   AATTC                                       285
Consensusaattc
```

Figure S1. Sequence alignment of original signal peptide (zα) and modified signal peptide (mα) using DNAMAN program.

46 codons in the modified signal peptide were optimized, resulting in changes of 50 nucleotides. G+C content increased from 41.1 to 47.0%. The modified signal peptide showed 82.5% identity with the original signal peptide (zα) after averaging the distribution of both the G+C content and the optimized codons.
