# Supplementary figures and images for: Improved production of a recombinant Rhizomucor miehei lipase expressed in Pichia pastoris and its application for conversion of microalgae oil to biodiesel
Source: Biotechnol Biofuels. 2014 Aug 4;7:111. doi: 10.1186/1754-6834-7-111 (PMC4364654; doi:10.1186/1754-6834-7-111)

Additional files 2: Figure S2

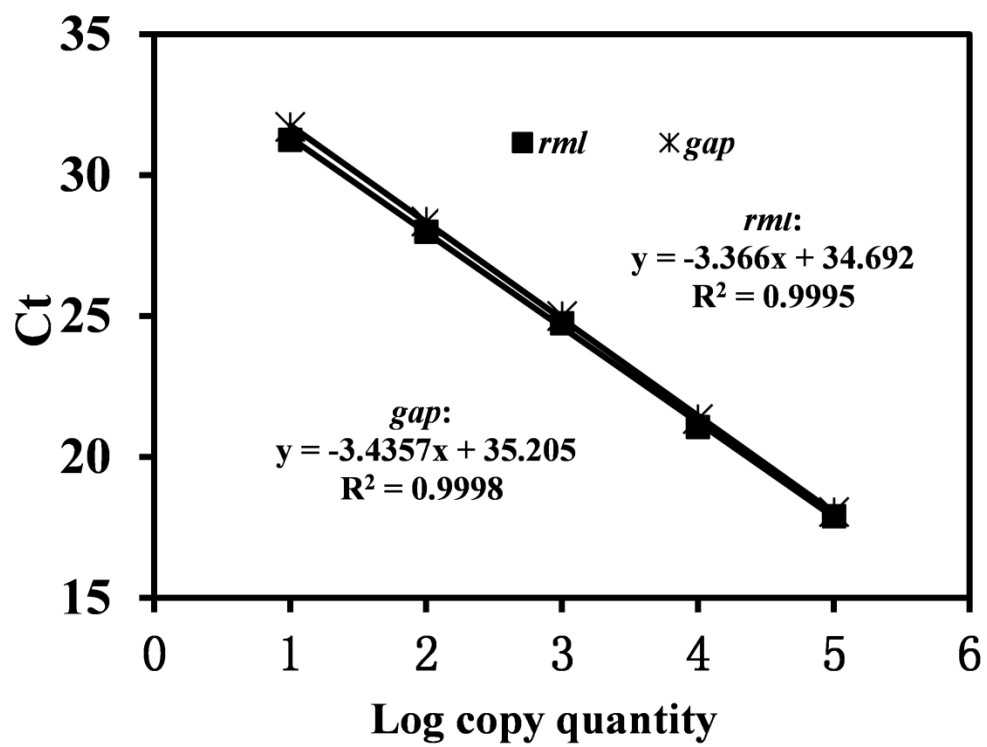

Figure S2. Standard curves of *gap* and *rml* detected by qPCR.

Supplement: Supplementary file 2 — Additional file 2: Figure S2: Standard curves of gap and rml detected by qPCR. (PDF 155 KB) [file 13068_2014_511_MOESM2_ESM.pdf]
