# Supplementary material for: Improved production of a recombinant Rhizomucor miehei lipase expressed in Pichia pastoris and its application for conversion of microalgae oil to biodiesel
Source: Biotechnol Biofuels. 2014 Aug 4;7:111. doi: 10.1186/1754-6834-7-111 (PMC4364654; doi:10.1186/1754-6834-7-111)
Supplement: Supplementary file 3 — Additional file 3: Table S1: Target gene copy number in four recombinant strains. (PDF 144 KB) [file 13068_2014_511_MOESM3_ESM.pdf]

Additional File 3: Table S1

Table S1. Target gene copy number in four recombinant strains.

Strain	Ct*(rml)*	Ct*(gap)*	Copy number
mα-1pRML-X33	20.33±0.11	20.64±0.08	1.06±0.04
mα-2pRML-X33	25.03±0.08	26.49±0.08	2.14±0.13
mα-4pRML-X33	17.74±0.11	20.04±0.03	4.17±0.15
mα-8pRML-X33	19.60±0.10	22.92±0.03	8.07±0.05
