# Supplementary material for: Improved production of a recombinant Rhizomucor miehei lipase expressed in Pichia pastoris and its application for conversion of microalgae oil to biodiesel
Source: Biotechnol Biofuels. 2014 Aug 4;7:111. doi: 10.1186/1754-6834-7-111 (PMC4364654; doi:10.1186/1754-6834-7-111)
Supplement: Supplementary file 4 — Additional file 4: Figure S3: Detection of glycosylation of lipases. Deglycosylation treatment using Endo Hf showed that pRML (Lipase GH2) was a glycosylated protein, whereas mRML had no glycosylation. Lane 1: protein markers (top to bottom: 100, 70, 55, 40, 35, 25, 15 kDa). Lane 2: Lipase GH2 without Endo Hf (no deglycosylation). Lane 3: Lipase GH2 with Endo Hf (with deglycosylation). Lane 4: mRML with Endo Hf (with deglycosylation). Lane 5: mRML without Endo Hf (no deglycosylation). (PDF 112 KB) [file 13068_2014_511_MOESM4_ESM.pdf]

Additional files 4:   Figure S3

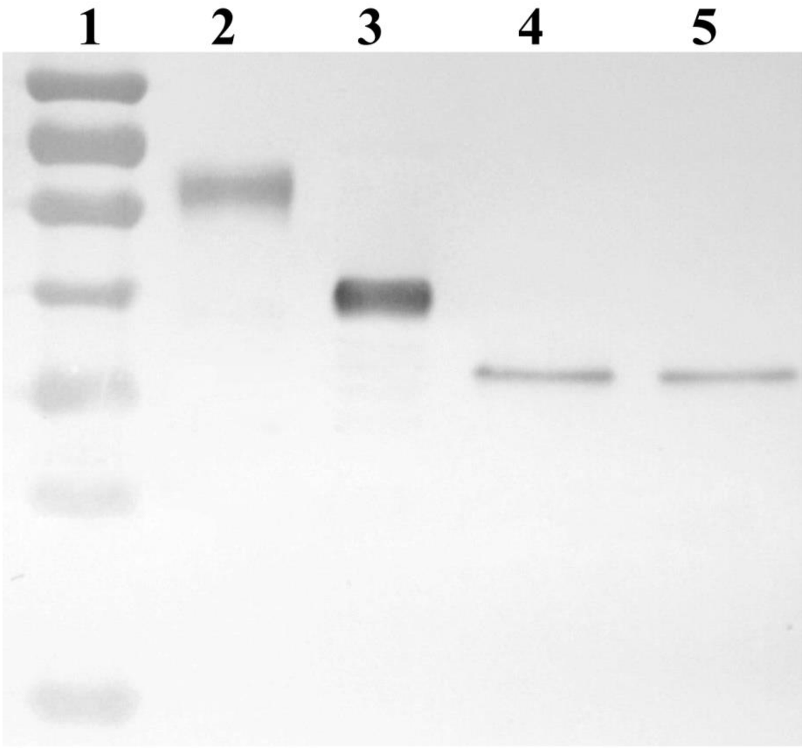

Figure S3. Detection of glycosylation of lipases.

Deglycosylation treatment using Endo Hf showed that pRML (Lipase GH2) was a glycosylated protein, whereas mRML had no glycosylation.

Lane 1: protein markers (top to bottom: 100, 70, 55, 40, 35, 25, 15 kDa). Lane 2: Lipase GH2 without Endo Hf (no deglycosylation). Lane 3: Lipase GH2 with Endo Hf (with deglycosylation). Lane 4: mRML with Endo Hf (with deglycosylation). Lane 5: mRML without Endo Hf (no deglycosylation).
