# Supplementary material for: Improved production of a recombinant Rhizomucor miehei lipase expressed in Pichia pastoris and its application for conversion of microalgae oil to biodiesel
Source: Biotechnol Biofuels. 2014 Aug 4;7:111. doi: 10.1186/1754-6834-7-111 (PMC4364654; doi:10.1186/1754-6834-7-111)
Supplement: Supplementary file 5 — Additional file 5: Table S2: Fatty acid analysis of microalgae oil by HPLC. (PDF 169 KB) [file 13068_2014_511_MOESM5_ESM.pdf]

Additional File 5: Table S2

Table S2. Fatty acid analysis of microalgae oil by HPLC.

**Fatty acid composition**	**Content (mg/g)**	**Fatty acid composition**	**Content (mg/g)**
**C6:0**	0.11 ±0.00	C8:0	1.16 ±0.15
**C10:0**	0.04 ±0.01	C12:0	0.10 ±0.00
**C13:0**	4.36 ±0.19	C14:0	5.56 ±0.46
**C15:0**	1.08 ±0.18	C16:0	117.55 ±8.50
**C16:1**	9.67 ±0.53	C17:0	1.67 ±0.14
**C18:0**	12.82 ±0.93	C18:1n9c	170.24 ±13.92
**C18:2n6c**	67.98 ±5.88	C18:3n6	12.01 ±1.04
**C18:3n3**	78.61 ±6.71	CLA-c9t11	16.97 ±1.44
**C20:0**	0.71 ±0.01	C20:1	2.38 ±0.21
**C20:4n6**	0.97 ±0.10	C20:5n3	0.25 ±0.03
**C22:0**	2.06 ±0.20	C23:0	0.20 ±0.19
**C22:1n9**	0.82 ±0.10	C24:0	0.91 ±0.46

Note: The microalgae oil extracted from 1 g microalgae powder contained 508.26 mg fatty acids, of which C16-C18 fatty acids comprised 487.52 mg (96% of the total)
