# Supplementary material for: Improved production of a recombinant Rhizomucor miehei lipase expressed in Pichia pastoris and its application for conversion of microalgae oil to biodiesel
Source: Biotechnol Biofuels. 2014 Aug 4;7:111. doi: 10.1186/1754-6834-7-111 (PMC4364654; doi:10.1186/1754-6834-7-111)
Supplement: Supplementary file 6 — Additional file 6: Table S3: Genes and primers used for qPCR. (PDF 183 KB) [file 13068_2014_511_MOESM6_ESM.pdf]

Additional File 6: Table S**3**

Table S**3.** Genes and primers used for qPCR.

Gene	GenBank accession number	Primer	Sequence 5′-3′	Amplicon size (bp)	Description[c]
**RML[a,b]**	**A02536.1**	***rml-f***<br>***rml-r***	**AGCATTGATGGTGGTATCCGC**<br>**ATAGATGAGCGTGCTCCAAGT**	**179**	***Rhizomucor miehei*** **lipase**
**GAP[a,b]**	**PPU62648**	***gap*-f**<br>***gap*-r**	**TACGTCATTGAGTCCACCGGT**<br>**TGGTAGTACAAGAAGCATTGGAG**	**184**	**glyceraldehyde-3-phosphate dehydrogenase**
**HAC1[b]**	**FR839628**	**HAC1-f**<br>**HAC1-r**	**CAAGAATCAGCCAAAGCC**<br>**TGCGAGTGGATGTAGATGC**	**199**	**bZIP transcription factor that regulates UPR**
**PDI[b]**	**ACF17572**	**PDI-f**<br>**PDI-r**	**GAGCAACAAGAAGTTTGGAGTTCC**<br>**CCTCATAAGCAGGAGCCATTC**	**260**	**Protein disulfide isomerase (PDI)**
**ERO1[b]**	**XP_002489645**	**ERO1-f**<br>**ERO1-r**	**CGTTAGCAAACCCTCAAATCC**<br>**GCAGAATCCCTCATCACCATT**	**201**	**PDI oxidase; glycoprotein required for oxidative protein folding in the ER, protein thiol-disulfide exchange**
**KAR2[b]**	**XM_002490982**	**KAR2-f**<br>**KAR2-r**	**CCTACTTCAACGACGCTCAA**<br>**CCACCCTCAATAGAAAGCAGA**	**199**	**BiP, a major Hsp70 chaperone in the ER (Sc), an ATPase regulates UPR; involved in ER quality control and ER-associated degradation**
**BFR2[b]**	**CCA39876**	**BFR2-f**<br>**BFR2-r**	**TGTGAGCCAAGAGTTTCCC**<br>**TGTCTGCCAAATAGTTGTCCAC**	**227**	**ER-to-Golgi transport, rDNA processing (Sc)**
**PMR1[b]**	**DQ239958**	**PMR1-f**<br>**PMR1-r**	**TTCACGCTTCCCACTTGA**<br>**TTGGCTGTTCCGATGACA**	**187**	**Ca^{2+} and Mg^{2+} transport in ATPase**
**SSO2[b]**	**FR839628**	**SSO2-f**<br>**SSO2-r**	**GTTCTCAACGGCTTTGCT**<br>**TGTCTACCAAGTTCTCGGTCT**	**194**	**Secretory vesicle fusion with plasma membrane**
**MON2[b]**	**FR839629**	**MON2-f**<br>**MON2-r**	**GGGACTCTTTGGGTATTTCG**<br>**ATCGGGCTCACCATCTTA**	**249**	**Golgi-to-endosome traffic, endocytosis, vacuole integrity**
**VPS10[b]**	**FR839629**	**VPS10-f**<br>**VPS10-r**	**TGAAGAATGGACGGGAAG**<br>**GCTCACATCGGTTAGACTCTG**	**221**	**Vacuolar protein sorting in Golgi membrane, late-Golgi**
**IMH1[b]**	**CCA37012**	**IMH1-f**<br>**IMH1-r**	**CGGATTCCAGTTTACTAAGCGA**<br>**AGGGTGTAGCAGATGAGCGA**	**219**	**Protein involved in vesicular transport; mediates transport between an endosomal compartment and Golgi; contains a Golgi localization (GRIP) domain**
**SSE1[b]**	**CCA37673**	**SSE1-f**<br>**SSE1-r**	**TGGACGAACTCTAAGTGTAGGG**<br>**CAAGGCTGAAGATGTGGAC**	**184**	**Nuclease exchange factors for HSP70 chaperones, ATPase of the Hsp90 chaperone complex; binds unfolded proteins; localized in cytoplasm**
**SEC31[b]**	**CCA37204**	**SEC31-f**<br>**SEC31-r**	**CGTGTTCCTCAATCAGCCA**<br>**TAGTTGCCTGGGTGAAGCA**	**198**	**COPII coat of secretory pathway vesicle component (p150), involved in protein transport from ER to Golgi; structural molecule**
**AQR1[b]**	**CCA37767**	**AQR1-f**<br>**AQR1-r**	**GCACCAGCAGTTTCCATAG**<br>**GAATACCAACATACCCAGGACT**	**201**	**Plasma membrane multidrug transporter of the major facilitator superfamily**

Primers of PDI and ERO1 as described by Sha et al. (2013). [a]Primers used for gene copy number quantification. [b]Primers used for transcription analysis. [c] As described by Gasser et al. (2007) and Idiris et al. (2010).
